# The impact of different endometrial preparation protocols on obstetric and neonatal complications in frozen-thawed embryo transfer: a retrospective cohort study of 3,458 singleton deliveries

**DOI:** 10.1186/s12958-022-01009-x

**Published:** 2022-09-22

**Authors:** Junting Xu, Hong Zhou, Tianfan Zhou, Yi Guo, Shanshan Liang, Yanping Jia, Kunming Li, Xiaoming Teng

**Affiliations:** 1 Centre for Reproductive Medicine, Shanghai First Maternity and Infant Hospital, School of Medicine, Tongji University, Shanghai, 200092 China; 2 Shanghai Key Laboratory of Maternal Fetal Medicine, Shanghai First Maternity and Infant Hospital, School of Medicine, Tongji University, Shanghai, 200092 China; 3 Clinical and Translational Research Center of Shanghai First Maternity and Infant Hospital, Shanghai Key Laboratory of Signaling and Disease Research, School of Life Sciences and Technology, Tongji University, Shanghai, 200092 China

**Keywords:** Endometrial preparation protocol, Frozen-thawed embryo transfer, Obstetric complication, Neonatal complication

## Abstract

**Background:**

Frozen-thawed embryo transfer (FET) is thought to be associated with obstetric and neonatal complications after in vitro fertilization/intracytoplasmic single sperm injection (IVF/ICSI) treatment. The study aimed to determine whether the endometrial preparation protocol is an influencing factor for these complications.

**Methods:**

We conducted a retrospective cohort study of 3,458 women who had singleton deliveries after IVF/ICSI–FET treatment at the Centre for Reproductive Medicine of Shanghai First Maternity and Infant Hospital between July 2016 and April 2021. The women were divided into three groups according to the endometrial preparation protocols: 2,029 women with programmed cycles, 959 with natural cycles, and 470 with minimal ovarian stimulation cycles. The primary outcomes were the incidence rates of obstetric and neonatal complications, namely, hypertensive disorders of pregnancy (HDP), gestational diabetes mellitus (GDM), intrahepatic cholestasis of pregnancy (ICP), placenta previa, preterm rupture of membranes (PROM), preterm delivery, postpartum haemorrhage, large for gestational age (LGA), small for gestational age (SGA), and macrosomia.

**Results:**

After adjustments for confounding variables by multivariate logistic regression analysis, the results showed that programmed cycles had an increased risk of HDP (aOR = 1.743; 95% CI, 1.110–2.735; *P* = 0.016) and LGA (aOR = 1.269; 95% CI, 1.011–1.592; *P* = 0.040) compared with natural cycles. Moreover, programmed cycles also increased the risk of LGA (aOR = 1.459; 95% CI, 1.083–1.965; *P* = 0.013) but reduced the risk of SGA (aOR = 0.529; 95% CI, 0.348–0.805; *P* = 0.003) compared with minimal ovarian stimulation cycles. There were no significant differences between natural cycles and minimal ovarian stimulation cycles.

**Conclusions:**

During IVF/ICSI–FET treatment, the risk of HDP and LGA was increased in women with programmed cycles. Therefore, for patients with thin endometrium, irregular menstruation or no spontaneous ovulation, minimal ovarian stimulation cycles may be a relatively safer option than programmed cycles.

**Supplementary Information:**

The online version contains supplementary material available at 10.1186/s12958-022-01009-x.

## Background

Over the last decade, the use of frozen-thawed embryo transfer (FET) cycles in assisted reproductive technology (ART) has dramatically risen [1]. According to the US Centers for Disease Control and Prevention (CDC), the number of FET cycles increased by 443.9% from 2010 to 2019, outpacing the increasing rate of fresh embryo transfer (ET) cycles. Major factors contributing to this trend are the implementation of vitrification techniques with excellent survival rates and the avoidance of the negative effects of supraphysiologic oestradiol (E_2_) and progesterone levels on the endometrium due to controlled ovarian stimulation (COS) in ET cycles [2–4]. Numerous studies have shown that compared with ET cycles, FET cycles are associated with increased maternal safety, improved pregnancy rates, decreased ectopic pregnancy rates, fewer complications (e.g., prevention of ovarian hyperstimulation syndrome, lower rates of antepartum haemorrhage) and better neonatal outcomes, including higher birth weight and lower risk of perinatal death [5–8]. However, emerging evidence suggests an increased risk of hypertensive disorders of pregnancy (HDP) in FET cycles [9]. Other obstetrical and neonatal complications, including postpartum haemorrhage, large for gestational age (LGA) and macrosomia, have also been reported to be increased in FET cycles [10–13]. However, it is still unclear what influencing factors in FET cycles may be related to these obstetrical and neonatal complications.

Recent studies have demonstrated that obstetric and neonatal complications in FET may be associated with the endometrial preparation protocol [14–17]. Compared to the natural cycle, the programmed cycle does not create a corpus luteum (CL), yet the minimal ovarian stimulation cycle produces one or more corpus lutea, which increases the risk of OHSS. The CL secretes oestrogen and progesterone, which play an important role in maintaining endometrial metaphase, regulating the immune response, suppressing the inflammatory response and improving blood circulation to the uteroplacenta. In addition, progesterone is involved in the process of extravillous trophoblast (EVT) cell invasion and vascular remodelling so that low pressure, and high blood flow can be supplied to the placenta [18, 19]. This is essential to facilitate normal development of the placenta and the natural growth of the foetus. Therefore, during ET, altered levels of steroid hormones in the body may lead to placenta-related complications such as HDP, placenta previa and placental abruption [18, 20].

Only a few studies have explored the association between endometrial preparation protocols and obstetric outcomes after FET. The number of patients included and the statistical method used in these studies were limited. Therefore, we performed this retrospective cohort study of 3,458 patients with singleton births after controlling for multiple pregnancy complication-related factors. This study aimed to provide up-to-date evidence to determine the differences in obstetrical and neonatal outcomes among patients who undergo different endometrial preparation protocols.

## Methods

### Study design and patients

This was a retrospective cohort study conducted at the Centre for Reproductive Medicine of Shanghai First Maternity and Infant Hospital comparing the obstetrical and neonatal outcomes among woman who underwent a programmed cycle, natural cycle and minimal ovarian stimulation cycle between July 2016 and April 2021. Our inclusion criteria were as follows: [1] patients under 43 years of age, [2] patients with a singleton birth, and [3] vitrified embryo(s) derived from the first IVF/ICSI cycles. The exclusion criteria were as follows: [1] cycles with preimplantation genetic testing (PGT), [2] patients with chronic hypertension or diabetes mellitus before the index pregnancy, or [3] patients with congenital or secondary uterine abnormalities (e.g., unicornuate uterus, didelphys uterine, septate uterus, adenomyosis, endometrial polyps, uterine fibroids, or intrauterine adhesions). This study was approved by the Research Ethics Committee of Shanghai First Maternity and Infant Hospital (No. KS22280).

### Ovarian stimulation and IVF/ICSI

All patients received COS treatment and monitoring and underwent oocyte collection, all of which were performed as described in detail previously [21, 22]. After follicular aspiration for approximately 4 to 6 h, oocytes were inseminated by conventional methods or ICSI, depending on sperm quality. Embryo culture and assessment were performed as described previously according to the number and regularity of blastomeres and the percentage and pattern of anucleate fragments [23]. High-quality embryos were frozen by vitrification on day 3, and other embryos were placed in extended culture, out of which good-morphologic-grade blastocysts were frozen on day 5. The vitrification procedure was performed using the Cryotop carrier system (Kitazato Biopharma Co., Tokyo, Japan) in combination with dimethylsulfoxide–ethylene–glycol–sucrose as cryoprotectants [24].

### Endometrial preparation and FET

At least 2 months after oocyte aspiration, the endometrium was prepared according to the three protocols as follows, at the discretion of local investigators. In the programmed cycle, oral oestradiol valerate (Progynova, Delpharm) was given at a dose of 4–6 mg daily on days 2–3 of the menstrual cycle. In the natural cycle, preparation occurred without any exogenous hormones and based on the endogenous luteinizing hormone surge observed during ovulation monitoring of the patient by transvaginal ultrasound beginning on day 10 of the menstrual cycle until ovulation. In the minimal ovarian stimulation cycle, preparation began by injection of hMG between days 3–5 of the menstrual cycle at a daily dose of 75 IU for the first 5 days. The daily dose of hMG ranged from 75 to 150 IU, depending on the response of the follicle to hMG. When the dominant follicle reached a diameter of ≥ 18 mm by transvaginal ultrasound examinations, hCG (Lizhu, China) 5,000 IU was injected to trigger final maturation of oocytes. A maximum of two high-quality embryos were thawed on the day of ET, which was 3 or 5 days after progesterone initiation. The thawing procedure following embryo vitrification was as described previously [22]. Only embryos in which more than 50% of the blastomeres were intact after thawing were transferred in FET cycles. Two hours after thawing, surviving embryos were transferred into the uterus under ultrasonographic guidance.

Oral dydrogesterone (Duphaston, Abbott Biologicals B.V.) 30 mg/d was added on the day of ovulation in the natural cycle or the minimal ovarian cycle. In the programmed cycle, when the endometrial thickness was ≥ 8 mm, dydrogesterone 40 mg/d and progesterone capsules (Utrogestan, Capsugel) 200 mg/d were added. Luteal-phase support was continued until 10 weeks of gestation.

### Outcome measures

A pregnancy test was performed 2 weeks after ET. Patients who had a positive pregnancy test underwent transvaginal ultrasound 2 weeks later to confirm a clinical pregnancy, defined as the presence of a gestation sac. Ongoing pregnancy was confirmed approximately 6 to 8 weeks after ET. Subsequent prenatal examinations were carried out in the obstetrics department. The outcome information included obstetric complications, gestational weeks, delivery date, delivery modes, new-born sex and birth weight, neonatal diseases, treatment and prognosis. The above data were collected by trained nurses who contacted patient by telephone after delivery.

Live birth was defined as any viable baby delivered at ≥ 28 weeks of gestation. Obstetric complications included HDP, gestational diabetes mellitus (GDM), intrahepatic cholestasis of pregnancy (ICP), placenta previa, preterm rupture of membrane (PROM), preterm delivery and postpartum haemorrhage. Neonatal outcomes included LGA, small for gestational age (SGA), and macrosomia. The determination of LGA and SGA was based on the birthweight reference for Chinese populations adjusted for sex and gestational age [25]. All detailed definitions of these terminologies are provided in Table S 1 in the Supplementary Appendix.

### Statistical analysis

All statistical analyses were undertaken in R version 4.1.1 (R Development Core Team). Continuous data are expressed as the mean (SD), and one-way ANOVA or Welch's ANOVA was chosen depending on whether the data met homoscedasticity. Categorical data are expressed as frequencies and percentages, and the variables in these measures were compared between study groups using chi-square or Fisher’s exact tests. Statistical significance was further analysed by applying Bonferroni's correction for post hoc pairwise analysis. Univariate regression analyses of variables that were potential predictors of the risk of obstetric and neonatal complications were performed. Logistic mixed-model regression analysis incorporating restricted cubic splines (RCSs) was performed to calculate the odds ratios (ORs) and 95% confidence intervals (CIs) for associations between the endometrial preparation protocols and the risk of obstetric and neonatal complications (HDP, preterm delivery, LGA and SGA). Regarding obstetric outcomes, the multiple regression analyses were adjusted for the following covariates: maternal age, maternal body mass index (BMI), menstrual cycle, duration of infertility, parity, cause of infertility, ART method, culture duration, number of transferred embryos and endometrial thickness on transfer day. In addition to the adjustments for the above variables, preterm birth, HDP and GDM were included as covariates in the analysis of neonatal outcomes. Regarding duration of infertility and endometrial thickness on transfer day, RCSs were generated to adjust for the nonlinear relationship between these continuous variables and the outcomes. When modelling RCS, the knot location of the spline was tested according to Harrell’s recommendations [26], and model fitting was assessed on the basis of the Akaike information criterion (AIC). A P value of less than 0.05 was considered statistically significant.

## Results

During our study period, 3458 women who underwent IVF/ICSI–FET cycles, including 2029 women with programmed cycles, 959 with natural cycles, and 470 with minimal ovarian stimulation cycles, met our study criteria. The demographic and main treatment characteristics of the women with singleton deliveries are listed in Table 1. Maternal age, maternal BMI, menstrual cycle, endometrial thickness on transfer day, gestational weeks and birth weight were significantly different among the three protocols. There were no significant differences in maternal smoking, duration of infertility, parity, cause of infertility, ART method, culture duration, proportion of high-quality embryos or number of transferred embryos among the three protocols.

**Table 1:** The demographic and main treatment characteristics for woman with singleton deliveries

	**Programmed cycles**	**Natural cycles**	**Minimal ovarian stimulation cycles**	***P* value**
	***n* = 2,029**	***n* = 959**	***n* = 470**	
Maternal age (year)^ab^	31.66 ± 3.90	32.68 ± 3.84	31.62 ± 3.77	< 0.001^c^
Maternal age, n (%)^ab^				
< 38	1866 (92.0)	854 (89.1)	438 (93.2)	0.009^d^
≥ 38	163 (8.0)	105 (10.9)	32 (6.8)	
Maternal BMI (kg/m^2^)^ab^	22.09 ± 3.51	21.30 ± 2.74	22.17 ± 3.21	< 0.001^c^
Maternal BMI, n (%)^ab^				
< 28	1914 (94.3)	935 (97.5)	445 (94.7)	< 0.001^d^
≥ 28	115 (5.7)	24 (2.5)	25 (5.3)	
Menstrual Cycle^abe^				
Regular	1508 (74.3)	935 (97.5)	388 (82.6)	< 0.001^d^
Irregular	521 (25.7)	24 (2.5)	82 (17.4)	
Maternal smoking, n (%)				
No smoking	2027 (99.9)	956 (99.7)	469 (99.8)	NS^f^
Smoking	2 (0.1)	3 (0.3)	1 (0.2)	
Duration of infertility (years)	3.14 ± 2.30	3.09 ± 2.19	3.12 ± 2.28	NS^c^
Parity, n (%)				
First	1902 (93.7)	885 (92.3)	437 (93.0)	NS^d^
High order	127 (6.3)	74 (7.7)	33 (7.0)	
Cause of infertility, n (%)				
Tubal factor	1007 (49.6)	496 (51.7)	265 (56.4)	NS^d^
Male factor	455 (22.4)	217 (22.6)	84 (17.9)	
Unexplained infertility	139 (6.9)	71 (7.4)	32 (6.8)	
Multiple factors	344 (17.0)	130 (13.6)	74 (15.7)	
Others	84 (4.1)	45 (4.7)	15 (3.2)	
ART method, n (%)				
IVF	1276 (62.9)	608 (63.4)	279 (59.4)	NS^d^
ICSI	753 (37.1)	351 (36.6)	191 (40.6)	
Culture duration, n (%)				
Cleavage-stage embryo	1565 (77.1)	752 (78.4)	370 (78.7)	NS^d^
Blastocyst embryo	464 (22.9)	207 (21.6)	100 (21.3)	
Proportion of high-quality embryos, n (%)	2225 (68.0)	1058 (68.4)	512 (69.1)	NS^d^
No. of transferred embryos, n (%)				
1	784 (38.6)	372 (38.8)	199 (42.3)	NS^d^
2	1245 (61.4)	587 (61.2)	271 (57.7)	
Endometrial thickness on transfer day(mm)^ae^	9.77 ± 1.82	10.12 ± 1.98	10.26 ± 2.42	< 0.001^c^
Gestational weeks (week)^b^	38.48 ± 1.76	38.56 ± 1.44	38.32 ± 1.60	0.029^c^
Birthweight(g)^e^	3350.81 ± 519.22	3310.11 ± 468.51	3251.92 ± 504.79	< 0.001^c^

Values are presented as mean ± standard deviation or n (%)

*ANOVA* analysis of variance, *BMI* body mass index, *NS* not statistically significant, *ART* assisted reproductive technology, *IVF* in vitro fertilization, *ICSI* intracytoplasmic single sperm injection

^a^Statistically significant differences between programmed cycles and natural cycles

^b^Statistically significant differences between natural cycles and minimal ovarian stimulation cycles

^c^One-way ANOVA

^d^Pearson chi-square test

^e^Statistically significant differences between programmed cycles and minimal ovarian stimulation cycles

^f^Fisher’s exact test

The outcomes of the obstetric and neonatal complications among the women with singleton deliveries after undergoing different endometrial preparation protocols are presented in Table 2. Regarding obstetric complications, women who had undergone programmed cycles and minimal ovarian stimulation cycles had higher rates of HDP (5.7% and 5.3% vs. 2.8%, respectively; *P* = 0.003) and preterm delivery (7.8% and 8.5% vs. 5.2%, respectively; *P* = 0.017) than women had undergone natural cycles. There were no statistically significant differences in the rate of GDM, ICP, placenta previa, PROM or postpartum haemorrhage among the three protocols. Regarding neonatal complications, programmed cycles had a higher risk of LGA (19.1% vs. 15.0% and 13.2%, respectively; *P* = 0.001) than natural cycles or minimal ovarian stimulation cycles. Programmed cycles had a lower risk of SGA (4.2% vs. 7.4%, *P* = 0.011) than minimal ovarian stimulation cycles; however, there was no significant difference in SGA risk between either programmed or minimal ovarian stimulation cycles and natural cycles. Additionally, there was no significant difference in the incidence of macrosomia among the three protocols. Furthermore, the univariate regression analysis of the different endometrial preparation protocols in predicting the incidence of obstetric and neonatal complications is shown in Table 3.

**Table 2:** Obstetric and neonatal complications for singleton births based on endometrial preparation protocols

	**Programmed cycles**	**Natural cycles**	**Minimal ovarian stimulation cycles**	***P* value**
	***n* = 2,029**	***n* = 959**	***n* = 470**	
**Obstetric complications**				
HDP^a^	115 (5.7)	27 (2.8)	25 (5.3)	0.003^b^
GDM	28 (1.4)	11 (1.1)	9 (1.9)	NS^b^
ICP	16 (0.8)	7 (0.7)	1 (0.2)	NS^c^
Placenta previa	49 (2.4)	15 (1.6)	10 (2.1)	NS^b^
PROM	63 (3.1)	34 (3.5)	14 (3.0)	NS^b^
Preterm delivery^ad^	159 (7.8)	50 (5.2)	40 (8.5)	0.017^b^
Postpartum haemorrhage	30 (1.5)	6 (0.6)	2 (0.4)	NS^c^
**Neonatal complications**				
LGA^ae^	387 (19.1)	144 (15.0)	62 (13.2)	0.001^b^
SGA^e^	86 (4.2)	54 (5.6)	35 (7.4)	0.011^b^
Macrosomia	45 (2.2)	15 (1.6)	8 (1.7)	NS^b^

Values are presented as n (%)

*HDP* hypertensive disorders of pregnancy, *GDM* gestational diabetes mellitus, *ICP* intrahepatic cholestasis of pregnancy, *PROM* preterm rupture of membrane, *LGA* large for gestational age, *SGA* small for gestational age

^a^Statistically significant differences between programmed cycles and natural cycles

^b^Pearson chi-square test

^c^Fisher’s exact test

^d^Statistically significant differences between natural cycles and minimal ovarian stimulation cycles

^e^Statistically significant differences between programmed cycles and minimal ovarian stimulation cycles

**Table 3:** Univariate regression analysis of the different endometrial preparation protocols for obstetric and neonatal complications

	**Programmed cycles vs Natural cycles**		**Minimal ovarian stimulation cycles vs Natural cycles**		**Programmed cycles vs Minimal ovarian stimulation cycles**	
	**Crude OR (95%CI)**	***P* value**	**Crude OR (95% CI)**	***P* value**	**Crude OR (95% CI)**	***P* value**
**Obstetric complications**						
HDP	2.074 (1.354–3.177)	0.001^a^	1.939 (1.113–3.380)	0.020^a^	1.069 (0.686–1.668)	0.767
GDM	1.206 (0.598–2.433)	0.601	1.682 (0.692–4.089)	0.251	0.717 (0.336–1.529)	0.389
ICP	1.081 (0.443–2.636)	0.864	0.290 (0.036–2.364)	0.248	3.728 (0.493–28.179)	0.202
Placenta previa	1.557 (0.869–2.791)	0.137	1.368 (0.610–3.069)	0.447	1.138 (0.572–2.264)	0.712
PROM	0.872 (0.570–1.333)	0.526	0.835 (0.444–1.572)	0.577	1.044 (0.580–1.879)	0.887
Preterm delivery	1.546 (1.114–2.145)	0.009^a^	1.691 (1.099–2.603)	0.017^a^	0.914 (0.636–1.313)	0.627
Postpartum haemorrhage	2.384 (0.989–5.746)	0.053	0.679 (0.136–3.376)	0.636	3.512 (0.836–14.746)	0.086
**Neonatal complications**						
LGA	1.334 (1.082–1.644)	0.007^a^	0.860 (0.624–1.185)	0.357	1.551 (1.161–2.071)	0.003^a^
SGA	0.742 (0.523–1.019)	0.094	1.348 (0.868–2.094)	0.183	0.550 (0.366–0.826)	0.004^a^
Macrosomia	1.427 (0.792–2.574)	0.237	1.090 (0.459–2.589)	0.846	1.310 (0.613–2.798)	0.486

Values are presented as n (%) or OR (95%CI)

*OR* odds ratio, *CI* confidence interval, *HDP* hypertensive disorders of pregnancy, *GDM* gestational diabetes mellitus, *ICP* intrahepatic cholestasis of pregnancy, *PROM* preterm rupture of membrane, *LGA* large for gestational age, *SGA* small for gestational age

^a^*P* < 0.05

Then, we performed multivariate logistic regression incorporating RCSs to determine the association between endometrial preparation protocols and the above complications with differences (Table 4). We found that after adjustments for confounding variables, the results showed that programmed cycles had an increased risk of HDP (aOR = 1.743; 95% CI, 1.110–2.735; *P* = 0.016) and LGA (aOR = 1.269; 95% CI, 1.011–1.592; *P* = 0.040) compared with natural cycles. Moreover, programmed cycles also increased the risk of LGA (aOR = 1.459; 95% CI, 1.083–1.965; *P* = 0.013) but reduced the risk of SGA (aOR = 0.529; 95% CI, 0.348–0.805; *P* = 0.003) compared with minimal ovarian stimulation cycles. There were no significant differences between natural cycles and minimal ovarian stimulation cycles.

**Table 4:** Adjusted odds ratios of obstetric and neonatal complications by multivariate analysis of endometrial preparation protocols

	**Programmed cycles vs Natural cycles**		**Minimal ovarian stimulation cycles vs Natural cycles**		**Programmed cycles vs Minimal ovarian stimulation cycles**	
	**Adjusted OR (95%CI)**	***P* value**	**Adjusted OR (95%CI)**	***P* value**	**Adjusted OR (95%CI)**	***P* value**
**Obstetric complications**^**a**^						
HDP	1.743 (1.110–2.735)	0.016^c^	1.739 (0.981–3.084)	0.058	1.002 (0.634–1.582)	0.994
Preterm delivery	1.368 (0.966–1.936)	0.077	1.511 (0.962–2.372)	0.073	0.905 (0.618–1.325)	0.609
**Neonatal complications**^**b**^						
LGA	1.269 (1.011–1.592)	0.040^c^	0.870 (0.624–1.214)	0.413	1.459 (1.083–1.965)	0.013^c^
SGA	0.697 (0.478–1.014)	0.059	1.316 (0.830–2.084)	0.243	0.529 (0.348–0.805)	0.003^c^

Values are presented as n (%) or OR (95%CI)

*OR* odds ratio, *CI* confidence interval, *HDP* hypertensive disorders of pregnancy, *GDM* gestational diabetes mellitus, *LGA* large for gestational age, *SGA* small for gestational age

^a^Adjustment included maternal age, maternal BMI, menstrual cycle, duration of infertility, parity, cause of infertility, ART method, culture duration, number of transferred embryos and endometrial thickness on transfer day

^b^Adjustment included maternal age, maternal BMI, menstrual cycle, duration of infertility, parity, cause of infertility, ART method, culture duration, number of transferred embryos, endometrial thickness on transfer day, preterm birth, HDP and GDM

^c^*P* < 0.05

We further performed univariate and multivariate regression analyses of other variables that were also potential predictors of the risk of the above complications with differences, as shown in TableS2-S5. Maternal BMI (aOR = 3.714; 95% CI, 2.309–5.973; *P* < 0.001) was an increased risk of HDP (Table S2). There was a decreased risk of LGA in women with HDP (aOR = 0.578; 95% CI, 0.341–0.978; *P* = 0.041) and preterm delivery (aOR = 0.306; 95% CI, 0.179–0.524; *P* < 0.001) and an increased risk of LGA in women with maternal BMI (aOR = 1.740; 95% CI, 1.185–2.556; *P* = 0.005) and blastocyst ET (aOR = 1.507; 95% CI, 1.206–1.884; *P* < 0.001) (Table S3). Likewise, there was an increased risk of SGA in women with HDP (aOR = 1.997; 95% CI, 1.143–3.488; *P* = 0.015), preterm delivery (aOR = 1.993; 95% CI, 1.219–3.261; *P* = 0.006) and GDM (aOR = 3.251; 95% CI, 1.386–7.626; *P* = 0.007) (Table S4). We also found that maternal age (aOR = 1.904; 95% CI, 1.272–2.850; *P* = 0.002), maternal BMI (aOR = 1.827; 95% CI, 1.092–3.056; *P* = 0.022), multipara (aOR = 1.608; 95% CI, 1.032–2.503; *P* = 0.036) and blastocyst ET (aOR = 1.474; 95% CI, 1.069–2.032; *P* = 0.018) were positive predictors of preterm delivery (Table S5).

## Discussion

With the increasing number of FET cycles in recent years, increasing attention has been given to obstetric and neonatal complications after transfer. Our study evaluated the effect of endometrial preparation protocols on obstetric and neonatal complications in our reproductive centre, which included only singleton deliveries after the transfer of embryos that were previously vitrified. After adjustment for confounding variables by multivariate logistic regression analysis, we showed that the risk of HDP was higher in women who conceived in programmed cycles than in those who conceived in natural cycles. Moreover, we showed a tendency towards increasing birth weight from minimal ovarian stimulation cycles to natural cycles to programmed cycles, and the risk of LGA was significantly increased when programmed cycles were compared with the natural and minimal ovarian stimulation cycles. The risk of SGA was significantly lower in women who conceived in programmed cycles than in those who conceived in minimal ovarian stimulation cycles, but there was no difference between either the programmed or minimal ovarian stimulation cycles and natural cycles.

Our results of obstetric and neonatal complications after programmed cycles are consistent to some extent with recently published studies from Sweden and Japan [14, 15]. One study of a cohort from Sweden, suggested that compared to natural and stimulated frozen cycles, programmed frozen cycles were associated with a higher risk of HDP, postpartum haemorrhage, preterm delivery and macrosomia. A study in Japan also demonstrated that pregnancy conceived from programmed cycles is associated with a higher risk of HDP but a lower risk of GDM. However, there was no statistically significant difference in the rate of GDM, postpartum haemorrhage or macrosomia among the three cycles in our study. In these two studies with large cohorts, both included women who had two or more embryos transferred, and there was no limit to the number of ART cycles per woman. Moreover, their methods of embryo cryopreservation included slow freezing and vitrification. These differences may impact their results.

HDP is a group of common clinical complications during the perinatal period and a major cause of maternal morbidity and mortality [27, 28]. Studies have demonstrated that risk factors for HDP include maternal comorbidities, such as advanced maternal age, chronic hypertension and obesity, a family history of preeclampsia, nulliparity or multiple pregnancies, and previous preeclampsia or intrauterine foetal growth restriction [29–31]. Therefore, we included only singleton births born to women under 43 years of age without hypertension or diabetes and adjusted for most of the risk factors mentioned above that may affect perinatal outcomes. The clear aetiology and pathophysiology of HDP remain elusive. Preeclampsia is usually associated with placental hypoperfusion and ischaemia, which is caused mainly by remodelling disorders of uterine spiral arteries [29, 32, 33]. Oestrogen and progesterone are essential for the development of a normal placenta, and altered levels of these sex steroid hormones, as seen in the programmed FET cycles, may lead to placenta-related complications [19]. A self-control study by Zhang et al. found significantly higher levels of E_2_ and P (2.1- and 14.5-fold on average, respectively) in the endometrium of the programmed cycle than in that of the natural cycle [34]. Several studies comparing placentas from women with preeclampsia to those from women with normal pregnancy showed that there was increased production of progesterone in the preeclamptic group [35–37]. Moreover, a prospective study by Tamimi et al. found that increased serum progesterone in the early third trimester was associated with the later development of preeclampsia [38]. EVT cells from the placenta invade the uterine decidual spiral arterioles and mediate the remodelling of these vessels such that a low pressure, high blood flow can be supplied to the placenta. High levels of progesterone may be a negative regulator of EVT and lead to shallow trophoblast invasion, resulting in inadequate remodelling of uterine spiral arteries, shallow placental implantation and subsequent development of preeclampsia [19].

Recent evidence suggests that the absence of the CL could also be responsible for this increased risk of HDP [39]. In addition to producing steroid hormones, the CL is a major source of vasoactive and angiogenic regulatory substances, such as vascular endothelial growth factor (VEGF), relaxin, members of the transforming growth factor (TGF) family, angiopoietins and epidermal growth factor, which may optimize implantation and placentation [40–42]. Abnormalities in the balance of vasoactive factors has been suggested to be a possible mechanism in preeclampsia. Li et al. showed in a study that free biologically active VEGF was significantly reduced in the serum of preeclamptic pregnancies compared to that of normal pregnancies [43]. Relaxin is secreted solely from the CL during pregnancy [44]. It is a potent vasodilator that mediates circulatory changes, including increases in effective renal plasma flow (ERPF), cardiac output, and arterial compliance [45–47]. Our results consistently suggested that pregnant women undergoing minimal ovarian stimulation cycles who have one or more CLs have a lower rate of HDP closer to that of the natural cycle. However, studies on how the vasoactive substances produced by the CL maintain balance are still limited, and it is necessary to improve our understanding of the pathophysiology of HDP, as this will allow better clinical management of this serious disorder.

In addition to the above factors, the increased risk of HDP may be related to the characteristics of the patients in programmed cycles. In our study, the majority of patients undergoing programmed cycles were those with thin endometrium, irregular menstruation or without spontaneous ovulation. Analysis of the characteristics of the patients also showed a higher proportion of obesity and thinner endometrial thickness on the day of transplantation in women undergoing programmed cycles than in those undergoing the minimal ovarian stimulation or natural cycles, and these characteristics were associated with poor pregnancy outcomes. Adipose tissue is known to mediate the changes associated with metabolic syndrome [48–50]. The interplay of inflammation and maternal metabolic syndrome is a proposed theory for the development of HDP. This interplay then leads to an increase in oxidative stress, resulting in endothelial dysfunction, maternal organ hypoperfusion and finally HDP [51]. Abnormal uterine artery blood flow in the thin endometrium prevents the intrauterine environment from being maintained and increases the risk of HDP [52]. Studies have also suggested that a thin endometrium appears to be associated with an aberrantly activated inflammatory environment [53].

There are few available studies on the possible effects of endometrial preparation protocols in FET cycles on new-born birth weight. Multiple studies have demonstrated a higher incidence of LGA after programmed cycles, which agrees with our study [14, 17, 54]. In addition, we found that blastocyst ET was an independent risk for the occurrence of LGA compared to cleavage-stage ET. The sample size in this study was large, and we performed univariable and multivariate logistic regression incorporating RCSs to minimize the source of bias. Moreover, we included a homogenous group of patients restricted to those who underwent ART for the first time with a singleton delivery and whose maternal age was less than 43 years. This study excluded cycles with PGT, oocyte donation, and patients with uterine abnormalities. The current study has certain limitations because of its retrospective design. First, there may have been some selection biases in the grouping. For example, when clinicians chose protocols based on the patients' individual circumstances, the majority of patients undergoing minimal ovarian stimulation or programmed cycles were those with thin endometrium, irregular menstruation or without spontaneous ovulation. In addition, analysis of the patients’ characteristics showed a higher proportion of obesity in each of these two groups, and obesity itself is a risk factor for HDP occurrence. This also may have contributed to selection bias in the grouping. Second, since hCG was applied to trigger the final maturation of follicles in minimal ovarian stimulation cycles, though not in the programmed or natural cycles, this factor may contribute to bias in the results, given that most studies have demonstrated that hCG may influence the occurrence of HDP [55]. In addition, the number of cycles in the minimal stimulation cycle group was quite small compared to that in the programmed or natural cycle group, which may have introduced bias in distribution. Our findings are significant in view of the increasing use of FET cycles in recent decades, and the results indicate a link between the chosen protocol and perinatal outcomes. We therefore think that when possible, physicians could choose natural cycles or minimal ovarian stimulation cycles to prepare the endometrium for ET in order to reduce the risk of HDP and LGA when making clinical decisions.

## Conclusions

Our study shows that programmed cycles are associated with an increased risk of HDP and LGA in IVF/ICSI–FET treatment. There were no differences in obstetric or neonatal complications between natural cycles and minimal ovarian stimulation cycles. Due to the limitations of retrospective experiments, further prospective randomized trials are needed to determine whether the changes seen in the observational trials are indeed accurate. Therefore, for patients with thin endometrium, irregular menstruation or no spontaneous ovulation, minimal ovarian stimulation may be a safer option than programmed cycles. Clinicians should also remain aware of the possibility of complications above when women achieve pregnancy after programmed cycles.

## Supplementary Information

**Additional file 1: Table S1.** Definition of obstetric and neonatal complications. **Table S2.** Univariate and multivariate analysis of predictor variables for HDP. **Table S3.** Univariate and multivariate analysis of predictor variables for LGA. **Table S4.** Univariate and multivariate analysis of predictor variables for SGA. **Table S5.** Univariate and multivariate analysis of predictor variables for preterm delivery.

## Data Availability

The datasets used and/or analyzed during the current study are available from the corresponding author on reasonable request.

## Abbreviations

FET: Frozen-thawed embryo transfer
IVF/ICSI: In vitro fertilization/Intracytoplasmic single sperm injection
HDP: Hypertensive disorders of pregnancy
GDM: Gestational diabetes mellitus
ICP: Intrahepatic cholestasis of pregnancy
PROM: Preterm rupture of membrane
SGA: Small for gestational age
LGA: Large for gestational age
aOR: Adjusted odds ratio
ART: Assisted reproductive technologies
CDC: Centers for Disease Control and Prevention
ET: Embryo transfer
COS: Controlled ovarian stimulation
OHSS: Ovarian hyperstimulation syndrome
EVT: Extravillous trophoblast
PGT: Preimplantation genetic testing
ORs: Odds ratios
CIs: Confidence intervals
RCSs: Restricted cubic splines
BMI: Body mass index
AIC: Akaike information criterion
CL: Corpus luteum
VEGF: Vascular endothelial growth factor
ERPF: Effective renal plasma flow
