# Supplementary material for: The impact of different endometrial preparation protocols on obstetric and neonatal complications in frozen-thawed embryo transfer: a retrospective cohort study of 3,458 singleton deliveries
Source: Reprod Biol Endocrinol. 2022 Sep 22;20:141. doi: 10.1186/s12958-022-01009-x (PMC9494872; doi:10.1186/s12958-022-01009-x)
Supplement: Supplementary file 1 — Additional file 1: Table S1. Definition of obstetric and neonatal complications. Table S2. Univariate and multivariate analysis of predictor variables for HDP. Table S3. Univariate and multivariate analysis of predictor variables for LGA. Table S4. Univariate and multivariate analysis of predictor variables for SGA. Table S5. Univariate and multivariate analysis of predictor variables for preterm delivery. [file 12958_2022_1009_MOESM1_ESM.docx]

Table S1

| Definition of obstetric and neonatal complications | |
| --- | --- |
| Terms | Definition |
| gestational hypertension | Diagnosed after 20 weeks' gestation; systolic blood pressure ≥140 mmHg or diastolic pressure ≥ 90 mmHg on two occasions, 4 h apart |
|  |  |
|  |  |
| preeclampsia | Gestational hypertension and the coexistence of one or both of the  following new-onset conditions: proteinuria; other maternal organ dysfunction; uteroplacental dysfunction |
|  |  |
|  |  |
|  |  |
| Gestational diabetes mellitus | The 75-g oral glucose tolerance test (OGTT) was performed for at  24–28 weeks of gestation. The diagnosis of gestational diabetes is made when any of the following plasma glucose values are met or exceeded: fasting: 92 mg/dL (5.1 mmol/L); 1 h: 180 mg/dL (10.0 mmol/L); 2 h: 153 mg/dL (8.5 mmol/L) |
|  |  |
|  |  |
|  |  |
|  |  |
|  |  |
| Intrahepatic cholestasis of pregnancy | Pruritus of cholestasis, elevated fasting serum bile acids > 10 μmol/L with elevated serum transaminases and absence of other diseases that cause pruritus and jaundice |
|  |  |
|  |  |
|  |  |
| Preterm delivery | Occur at less than 37 weeks' gestational age |
|  |  |
| Postpartum haemorrhage | Blood loss of more than 500 mL from the female genital tract after  delivery of the fetus (or >1000 mL after a caesarean section) |
|  |  |
|  |  |
| Large for gestational age | Birth weight > 90th percentile for gestational age |
|  |  |
| Small for gestational age | Birth weight < 10th percentile for gestational age |
|  |  |
| Macrosomia | Birth weight > 4000 g |

Table S2

| Univariate and multivariate analysis of predictor variables for HDP | | | | | | |
| --- | --- | --- | --- | --- | --- | --- |
| **Predictor variable** | |  | **Crude OR (95% CI)** | **P-Value** | **Adjusted OR (95%CI)** | **P-Value** |
| Maternal age | |  |  |  |  |  |
|  | <38 |  | 1.000 |  | 1.000 |  |
|  | ≥38 |  | 1.195 (0.713-2.003) | 0.498 | 1.384 (0.799-2.400) | 0.247 |
| Maternal BMI | |  |  |  |  |  |
|  | <28 |  | 1.000 |  | 1.000 |  |
|  | ≥28 |  | 4.213 (2.682-6.619) | <0.001^a^ | 3.714 (2.309-5.973) | <0.001^a^ |
| Menstrual cycle | |  |  |  |  |  |
|  | Regular |  | 1.000 |  | 1.000 |  |
|  | Irregular |  | 1.773 (1.248-2.519) | 0.001^a^ | 1.151 (0.773-1.715) | 0.488 |
| Parity | |  |  |  |  |  |
|  | First |  | 1.000 |  | 1.000 |  |
|  | High order |  | 0.501 (0.219-1.144) | 0.101 | 0.490 (0.212-1.130) | 0.094 |
| Cause of infertility | |  |  |  |  |  |
|  | Tubal factor |  | 1.000 |  | 1.000 |  |
|  | Male factor |  | 0.938 (0.621-1.419) | 0.763 | 0.865 (0.551-1.358) | 0.528 |
|  | Unexplained infertility | | 0.979 (0.514-1.864) | 0.949 | 0.983 (0.509-1.897) | 0.958 |
|  | Multiple factors | | 1.532 (1.030-2.279) | 0.035^a^ | 1.329 (0.868-2.033) | 0.190 |
|  | Others |  | 0.437 (0.136-1.402) | 0.164 | 0.545 (0.125-2.376) | 0.419 |
| ART method | |  |  |  |  |  |
|  | IVF |  | 1.000 |  | 1.000 |  |
|  | ICSI |  | 1.126 (0.819-1.546) | 0.465 | 1.161 (0.815-1.654) | 0.409 |
| Frozen embryo transfer protocol | | |  |  |  |  |
|  | Programmed cycles | | 1.000 |  | 1.000 |  |
|  | Natural cycles | | 0.482 (0.314-0.739) | <0.001^a^ | 0.574 (0.366-0.901) | 0.016^a^ |
|  | Minimal ovarian stimulation cycles | | 0.935 (0.599-1.459) | 0.767 | 0.998 (0.632-1.576) | 0.994 |
| Culture duration | |  |  |  |  |  |
|  | Cleavage-stage embryo | | 1.000 |  | 1.000 |  |
|  | Blastocyst embryo | | 0.852 (0.577-1.258) | 0.420 | 0.795 (0.522-1.210) | 0.284 |
| No. of transferred embryos | | |  |  |  |  |
|  | 1 | | 1.000 |  | 1.000 |  |
|  | 2 | | 0.865 (0.631-1.184) | 0.367 | 0.723 (0.515-1.016) | 0.062 |
| *Note*: Values are presented as n (%) or OR (95%CI). OR = odds ratio; CI = confidence interval; BMI = body mass index; ART = assisted reproductive technology; IVF = in vitro fertilization; ICSI = intracytoplasmic sperm injection.  For variables of duration of infertility and endometrial thickness on transfer day, restricted cubic splines were generated to adjust for the nonlinear relationship between these continuous variables and HDP. Thus, quantitative results were not presented.  ^a^P<0.05. | | | | | | |

Table S3

| Univariate and multivariate analysis of predictor variables for LGA | | | | | | |
| --- | --- | --- | --- | --- | --- | --- |
| **Predictor variable** | |  | **Crude OR (95% CI)** | **P-Value** | **Adjusted OR (95%CI)** | **P-Value** |
| Maternal age | |  |  |  |  |  |
|  | <38 |  | 1.000 |  | 1.000 |  |
|  | ≥38 |  | 0.905 (0.656-1.249) | 0.545 | 0.991 (0.695-1.411) | 0.959 |
| Maternal BMI | |  |  |  |  |  |
|  | <28 |  | 1.000 |  | 1.000 |  |
|  | ≥28 |  | 1.656 (1.149-2.385) | 0.007^a^ | 1.740 (1.185-2.556) | 0.005^a^ |
| Menstrual cycle | |  |  |  |  |  |
|  | Regular |  | 1.000 |  | 1.000 |  |
|  | Irregular |  | 1.371 (1.104-1.702) | 0.004^a^ | 1.261 (0.991-1.605) | 0.059 |
| Parity | |  |  |  |  |  |
|  | First |  | 1.000 |  | 1.000 |  |
|  | High order |  | 1.269 (0.912-1.765) | 0.158 | 1.380 (0.980-1.942) | 0.065 |
| Cause of infertility | |  |  |  |  |  |
|  | Tubal factor |  | 1.000 |  | 1.000 |  |
|  | Male factor |  | 0.898 (0.713-1.131) | 0.360 | 0.905 (0.700-1.170) | 0.446 |
|  | Unexplained infertility | | 0.971 (0.679-1.388) | 0.871 | 1.001 (0.692-1.448) | 0.994 |
|  | Multiple factors | | 1.088 (0.849-1.394) | 0.503 | 1.030 (0.791-1.343) | 0.825 |
|  | Others |  | 0.904 (0.570-1.437) | 0.671 | 0.895 (0.431-1.859) | 0.766 |
| ART method | |  |  |  |  |  |
|  | IVF |  | 1.000 |  | 1.000 |  |
|  | ICSI |  | 0.932 (0.775-1.120) | 0.452 | 0.944 (0.764-1.166) | 0.595 |
| Frozen embryo transfer protocol | | |  |  |  |  |
|  | Programmed cycles | | 1.000 | 1.000 | 1.000 |  |
|  | Natural cycles | | 0.750 (0.608-0.924) | 0.007^a^ | 0.788 (0.628-0.989) | 0.040^a^ |
|  | Minimal ovarian stimulation cycles | | 0.644 (0.483-0.861) | 0.003^a^ | 0.686 (0.509-0.924) | 0.013^a^ |
| Culture duration | |  |  |  |  |  |
|  | Cleavage-stage embryo | | 1.000 |  | 1.000 |  |
|  | Blastocyst embryo | | 1.452 (1.188-1.774) | <0.001^a^ | 1.507 (1.206-1.884) | <0.001^a^ |
| No. of transferred embryos | | |  |  |  |  |
|  | 1 | | 1.000 |  | 1.000 |  |
|  | 2 | | 0.994 (0.830-1.192) | 0.953 | 1.144 (0.934-1.401) | 0.192 |
| Preterm delivery | |  |  |  |  |  |
|  | No |  | 1.000 |  | 1.000 |  |
|  | Yes |  | 0.292 (0.172-0.496) | <0.001^a^ | 0.306 (0.179-0.524) | <0.001^a^ |
| HDP | | |  |  |  |  |
|  | No |  | 1.000 |  | 1.000 |  |
|  | Yes |  | 0.534 (0.321-0.889) | 0.016^a^ | 0.578 (0.341-0.978) | 0.041^a^ |
| GDM | |  |  |  |  |  |
|  | No |  | 1.000 |  | 1.000 |  |
|  | Yes |  | 0.966 (0.450-2.074) | 0.929 | 0.970 (0.441-2.131) | 0.939 |
| *Note*: Values are presented as n (%) or OR (95%CI). OR = odds ratio; CI = confidence interval; BMI = body mass index; ART = assisted reproductive technology; IVF = in vitro fertilization; ICSI = intracytoplasmic sperm injection; HDP = hypertensive disorders of pregnancy; GDM = gestational diabetes mellitus.  For variables of duration of infertility and endometrial thickness on transfer day, restricted cubic splines were generated to adjust for the nonlinear relationship between these continuous variables and LGA. Thus, quantitative results were not presented.  ^a^P<0.05. | | | | | | |

Table S4

| Univariate and multivariate analysis of predictor variables for SGA | | | | | | |
| --- | --- | --- | --- | --- | --- | --- |
| **Predictor variable** | |  | **Crude OR (95% CI)** | **P-Value** | **Adjusted OR (95%CI)** | **P-Value** |
| Maternal age | |  |  |  |  |  |
|  | <38 |  | 1.000 |  | 1.000 |  |
|  | ≥38 |  | 0.831(0.467-1.481) | 0.531 | 0.757 (0.411-1.397) | 0.374 |
| Maternal BMI | |  |  |  |  |  |
|  | <28 |  | 1.000 |  | 1.000 |  |
|  | ≥28 |  | 0.960 (0.464-1.987) | 0.913 | 0.843 (0.394-1.804) | 0.660 |
| Menstrual cycle | |  |  |  |  |  |
|  | Regular |  | 1.000 |  | 1.000 |  |
|  | Irregular |  | 0.853 (0.564-1.291) | 0.453 | 0.855 (0.542-1.348) | 0.499 |
| Parity | |  |  |  |  |  |
|  | First |  | 1.000 |  | 1.000 |  |
|  | High order |  | 0.648 (0.314-1.334) | 0.239 | 0.635 (0.304-1.325) | 0.226 |
| Cause of infertility | |  |  |  |  |  |
|  | Tubal factor |  | 1.000 |  | 1.000 |  |
|  | Male factor |  | 1.252 (0.866-1.809) | 0.232 | 1.327 (0.884-1.992) | 0.173 |
|  | Unexplained infertility | | 0.920 (0.484-1.749) | 0.799 | 0.937 (0.488-1.800) | 0.846 |
|  | Multiple factors | | 0.924 (0.586-1.456) | 0.732 | 0.885 (0.544-1.441) | 0.623 |
|  | Others |  | 0.840 (0.361-1.956) | 0.686 | 0.613 (0.145-2.588) | 0.505 |
| ART method | |  |  |  |  |  |
|  | IVF |  | 1.000 |  | 1.000 |  |
|  | ICSI |  | 1.120 (0.821-1.528) | 0.474 | 0.983 (0.691-1.399) | 0.925 |
| Frozen embryo transfer protocol | | |  |  |  |  |
|  | Programmed cycles | | 1.000 |  | 1.000 |  |
|  | Natural cycles | | 1.348 (0.951-1.912) | 0.094 | 1.436 (0.986-2.090) | 0.059 |
|  | Minimal ovarian stimulation cycles | | 1.818 (1.211-2.730) | 0.004^a^ | 1.889 (1.243-2.871) | 0.003^a^ |
| Culture duration | |  |  |  |  |  |
|  | Cleavage-stage embryo | | 1.000 |  | 1.000 |  |
|  | Blastocyst embryo | | 0.623 (0.410-0.946) | 0.026^a^ | 0.635 (0.403-1.001) | 0.050 |
| No. of transferred embryos | | |  |  |  |  |
|  | 1 | | 1.000 |  | 1.000 |  |
|  | 2 | | 1.216 (0.884-1.672) | 0.229 | 1.075 (0.759-1.524) | 0.684 |
| Preterm delivery | |  |  |  |  |  |
|  | No |  | 1.000 |  | 1.000 |  |
|  | Yes |  | 2.160 (1.375-3.393) | <0.001^a^ | 1.993 (1.219-3.261) | 0.006^a^ |
| HDP | | |  |  |  |  |
|  | No |  | 1.000 |  | 1.000 |  |
|  | Yes |  | 2.247 (1.328-3.804) | 0.003^a^ | 1.997 (1.143-3.488) | 0.015^a^ |
| GDM | |  |  |  |  |  |
|  | No |  | 1.000 |  | 1.000 |  |
|  | Yes |  | 3.884 (1.790-8.430) | <0.001^a^ | 3.251 (1.386-7.626) | 0.007^a^ |
| *Note*: Values are presented as n (%) or OR (95%CI). OR = odds ratio; CI = confidence interval; BMI = body mass index; ART = assisted reproductive technology; IVF = in vitro fertilization; ICSI = intracytoplasmic sperm injection; HDP = hypertensive disorders of pregnancy; GDM = gestational diabetes mellitus.  For variables of duration of infertility and endometrial thickness on transfer day, restricted cubic splines were generated to adjust for the nonlinear relationship between these continuous variables and SGA. Thus, quantitative results were not presented.  ^a^P<0.05. | | | | | | |

Table S5

| Univariate and multivariate analysis of predictor variables for preterm delivery | | | | | | |
| --- | --- | --- | --- | --- | --- | --- |
| **Predictor variable** | |  | **Crude OR (95% CI)** | **P-Value** | **Adjusted OR (95%CI)** | **P-Value** |
| Maternal age | |  |  |  |  |  |
|  | <38 |  | 1.000 |  | 1.000 |  |
|  | ≥38 |  | 2.009 (1.391-2.902) | <0.001^a^ | 1.904 (1.272-2.850) | 0.002^a^ |
| Maternal BMI | |  |  |  |  |  |
|  | <28 |  | 1.000 |  | 1.000 |  |
|  | ≥28 |  | 1.746 (1.062-2.868) | 0.028^a^ | 1.827 (1.092-3.056) | 0.022^a^ |
| Menstrual cycle | |  |  |  |  |  |
|  | Regular |  | 1.000 |  | 1.000 |  |
|  | Irregular |  | 1.307 (0.956-1.787) | 0.093 | 1.117 (0.785-1.589) | 0.540 |
| Parity | |  |  |  |  |  |
|  | First |  | 1.000 |  | 1.000 |  |
|  | High order |  | 1.682 (1.094-2.585) | 0.018^a^ | 1.608 (1.032-2.503) | 0.036^a^ |
| Cause of infertility | |  |  |  |  |  |
|  | Tubal factor |  | 1.000 |  | 1.000 |  |
|  | Male factor |  | 0.712 (0.500-1.014) | 0.060 | 0.723 (0.493-1.062) | 0.098 |
|  | Unexplained infertility | | 0.781 (0.450-1.354) | 0.378 | 0.806 (0.460-1.410) | 0.449 |
|  | Multiple factors | | 0.930 (0.645-1.341) | 0.698 | 0.912 (0.620-1.342) | 0.641 |
|  | Others |  | 1.172 (0.646-2.127) | 0.601 | 0.782 (0.301-2.035) | 0.615 |
| ART method | |  |  |  |  |  |
|  | IVF |  | 1.000 |  | 1.000 |  |
|  | ICSI |  | 0.977 (0.748-1.276) | 0.865 | 1.078 (0.799-1.456) | 0.623 |
| Frozen embryo transfer protocol | | |  |  |  |  |
|  | Programmed cycles | | 1.000 |  | 1.000 |  |
|  | Natural cycles | | 0.647 (0.466-0.898) | 0.009^a^ | 0.731 (0.517-1.035) | 0.077 |
|  | Minimal ovarian stimulation cycles | | 1.094 (0.762-1.572) | 0.627 | 1.105 (0.754-1.617) | 0.609 |
| Culture duration | |  |  |  |  |  |
|  | Cleavage-stage embryo | | 1.000 |  | 1.000 |  |
|  | Blastocyst embryo | | 1.310 (0.978-1.754) | 0.070 | 1.474 (1.069-2.032) | 0.018^a^ |
| No. of transferred embryos | | |  |  |  |  |
|  | 1 | | 1.000 |  | 1.000 |  |
|  | 2 | | 1.048 (0.804-1.366) | 0.729 | 1.084 (0.807-1.456) | 0.591 |
| *Note*: Values are presented as n (%) or OR (95%CI). OR = odds ratio; CI = confidence interval; BMI = body mass index; ART = assisted reproductive technology; IVF = in vitro fertilization; ICSI = intracytoplasmic sperm injection;  For variables of duration of infertility and endometrial thickness on transfer day, restricted cubic splines were generated to adjust for the nonlinear relationship between these continuous variables and preterm delivery. Thus, quantitative results were not presented.  ^a^P<0.05. | | | | | | |
